# Early Warning of Cucumber Angular Leaf Spot by Estimating Airborne Pathogen Aerosols with Particulate Matter Sensors

**DOI:** 10.3390/plants15162510

**Published:** 2026-08-20

**Authors:** Xin Li, Leng Han, Yuheng Xing, Yanxia Shi, Xuewen Xie, Lei Li, Tengfei Fan, Sheng Xiang, Xianhua Sun, Baoju Li, Ali Chai

**Affiliations:** 1 College of Plant Protection, Shenyang Agricultural University, Shenyang 110866, China; lixinsyau2015@163.com; 2 State Key Laboratory of Vegetable Biobreeding, Institute of Vegetables and Flowers, Chinese Academy of Agricultural Sciences, Beijing 100081, China; cauleng@163.com (L.H.);; 3 College of Mechanical and Electrical Engineering, Hebei Agricultural University, Baoding 071500, China

**Keywords:** cucumber angular leaf spot, pathogen aerosols, disease monitoring, particulate matter, disease index prediction, machine learning

## Abstract

Airborne bacterial diseases driven by pathogen aerosols in enclosed greenhouses spread rapidly, challenging traditional early-warning methods. This study developed a two-step monitoring system for cucumber angular leaf spot using low-cost particulate matter (PM) sensors, qPCR, and machine learning. Evaluated across spatially independent greenhouse trials using 732 plot-days of data, PM sensors were utilized as dynamic physical proxies alongside microclimate data. These proxies continuously estimated the fluctuations of pathogen aerosols suspended in the greenhouse air. When estimated aerosol risks exceeded a pathogenic threshold, targeted air sampling and qPCR quantification were triggered. For pathogen monitoring, the Extra Trees (ET) surveillance model accurately predicted the accumulation of airborne pathogen aerosols (R^2^ = 0.884). For disease forecasting, by integrating the quantified aerosol loads with environmental factors, the XGBoost prediction model forecasted the daily disease index change rate with high precision (R^2^ = 0.874). SHapley Additive exPlanations (SHAP) analysis confirmed that the concentration of airborne pathogen aerosols and vapor pressure deficit were primary drivers of disease expansion. By combining continuous physical sensing of greenhouse air with risk-triggered biological quantification, this framework provides a feasible strategy to partly compensate for the lack of biological specificity of PM sensors and supports early-warning management of airborne bacterial diseases in protected agriculture.

## 1. Introduction

Plant diseases are primary factors limiting yield and quality in protected agriculture. Airborne diseases pose a particularly serious challenge due to their rapid transmission, long-distance dispersal, and broad infection range. Compared with field conditions, protected agriculture enhances crop productivity through precise control of temperature, humidity, and other environmental factors. However, the enclosed structure, high air humidity, and limited airflow exchange within greenhouses create microclimate conditions that promote the accumulation and dispersal of airborne plant pathogens [1,2]. In recent years, numerous studies have demonstrated that in controlled environments, airborne transmission is a critical pathway for pathogen entry and dissemination. Fungal spores, bacterial aerosols, and pathogen-bearing particulate matter can be transported by airflow and rapidly spread within the greenhouse, generating new infection foci and markedly accelerating disease epidemics [3,4,5]. Bacterial diseases are particularly problematic, as their pathogens can be released into the air through splash dispersal, airflow disturbances, or cultivation activities, and subsequently redeposited onto leaf surfaces as aerosols, leading to rapid short-term disease spread [6,7]. With the expansion of protected agriculture, traditional disease management strategies that rely on symptom recognition or field scouting have become increasingly inadequate for the timely capture of early infection dynamics [8,9]. Since airborne diseases often complete initial infection before visible symptoms appear, early-warning systems must transition from symptom-based detection to predictive approaches that anticipate pathogen transmission.

Among various bacterial infections in vegetable production, angular leaf spot (*Pseudomonas amygdali* pv. *lachrymans*, *Pal*) is one of the most severe diseases. It commonly causes angular lesions, marginal necrosis, and premature yellowing, ultimately reducing photosynthetic leaf area, inducing fruit malformations, and significantly decreasing yield [10,11]. In controlled environments such as greenhouses, the enclosed space, poor air exchange, and high humidity, combined with high-density planting, facilitate the formation and dispersal of pathogen particles into the air, thereby rapidly triggering disease outbreaks [12,13]. Infected plants are known to release pathogen aerosols in the micrometer range, which can be transported by airflow and deposited onto healthy leaf surfaces, initiating new infections [14]. Temperature and humidity are key environmental factors affecting the release, survival, and infectivity of pathogen aerosols [14,15]. Under conditions of high humidity and suitable temperatures, both the quantity and viability of aerosols are elevated, thereby facilitating pathogen infection [15,16]. Airborne transmission allows pathogens to accumulate and spread in the air before symptoms appear, increasing the difficulty of early disease monitoring [7,17,18]. In addition, overhead irrigation, ventilation, and routine management practices in greenhouses can further promote the generation and dispersal of pathogen particles [19].

Current disease early-warning systems and pesticide application decisions largely rely on plant phenotypic recognition or environmental factor modelling. Traditional methods primarily assess disease occurrence by observing leaf color changes, lesion morphology, or wilting symptoms. However, in the early stages, symptoms are often subtle and nonspecific. Initial leaf lesions, chlorosis, or marginal necrosis caused by different foliar diseases can be easily confused, limiting the accuracy of image-based recognition methods during early infection stages [20,21]. Even when advanced techniques such as chlorophyll measurement or hyperspectral imaging enable early-stage disease diagnosis, these methods are costly and operationally complex, making large-scale deployment in greenhouse or other on-site environments challenging [22,23,24]. Meanwhile, models based on environmental factors can reveal overall trends of disease occurrence, but they lack direct insight into the dynamic changes in pathogens and can only indicate whether environmental conditions are conducive to disease spread and infection [25,26,27,28]. In contrast, nucleic acid detection techniques can directly quantify the presence and concentration of pathogens, serving as the “gold standard” for confirming pathogen load [29,30,31]. These techniques enable early and precise monitoring, even before visible symptoms develop, thereby supporting informed early-warning and intervention strategies.

The “disease triangle” is a fundamental theoretical framework for plant disease occurrence, which posits that disease development and epidemics require specific conditions in all three components: the host, the pathogen, and the environment [32]. The quantity, viability, and transmissibility of the pathogen directly determine the intensity and rate of disease development. In most early-warning models based on environmental data, analyses of the “disease triangle” typically focus on the host and environmental components, emphasizing whether conditions are favorable for disease progression, while the pathogen itself is rarely monitored or quantified dynamically [33,34,35]. However, factors such as fluctuations in airborne pathogen concentrations, generation and dispersal of pathogen particles, deposition, and infection efficiency play a decisive role in the spatial spread and rate of disease occurrence [36,37]. Therefore, establishing an integrated early-warning system that combines pathogen aerosol monitoring, nucleic acid quantification, and environmental factor modelling offers a novel approach for achieving early and quantitative disease prediction. However, direct continuous bio-monitoring in greenhouses remains prohibitively expensive. While low-cost laser scattering PM sensors are widely available, they lack biological specificity and cannot distinguish pathogen aerosols from inorganic dust.

In this study, an early-warning system for cucumber angular leaf spot was developed. Rather than relying on PM sensors for direct pathogen detection, this system utilizes them as continuous physical proxies to capture aerosol fluctuation risks under specific greenhouse microclimates, triggering precise qPCR quantification only when necessary. The main contributions of this work are: (1) incorporating pathogen-specific dynamic information into the predictive model via a two-step risk-triggered mechanism; (2) developing a low-cost laser scattering particulate matter sensor system to provide continuous surrogate monitoring of aerosol risks in protected agriculture.

## 2. Results

### 2.1. Dataset Characteristics

A total of 732 complete plot-days of monitoring data were collected in this study (aggregating daily observations across the 14 plots over the spring and autumn periods). Specifically, 14 spatially independent plots distributed across multiple greenhouses (plastic arch tunnels and solar greenhouses in different districts) were monitored simultaneously in each season, ensuring that the model training and validation datasets encompass diverse microclimatic conditions and physical structures without spatial autocorrelation. After filtering out incomplete daily sequences caused by temporary sensor downtime, approximately 26 valid monitoring days per plot were retained for each season (14 plots × ~26 days × 2 seasons ≈ 732 plot-days). No data augmentation or temporal interpolation was applied to the dataset, ensuring each plot-day represented an authentic spatial–temporal sample without data leakage. The dataset included 98 days from healthy plants, for which the pathogen aerosol concentration was set to 0, while the remaining samples exhibited varying degrees of disease index (DI). All data were divided into training and validation sets at a 7:3 ratio. The training set was further split into training and test subsets at an 8:2 ratio for hyperparameter optimisation. The validation set was reserved for final model performance evaluation. The overall temperature and humidity distributions were concentrated between 15 and 30 °C, with relative humidity mostly ranging from 30 to 90%, encompassing the meteorological characteristics of both spring and autumn seasons. According to previous pathological studies [14], healthy plants reach a peak in aerosol release 2–4 days after infection. The logarithm of aerosol concentration and the rate of change in disease index do not exhibit a strictly linear relationship. Some data points show negative disease index change rates because the growth of new cucumber leaves exceeds the infection rate.

**Figure 1:**
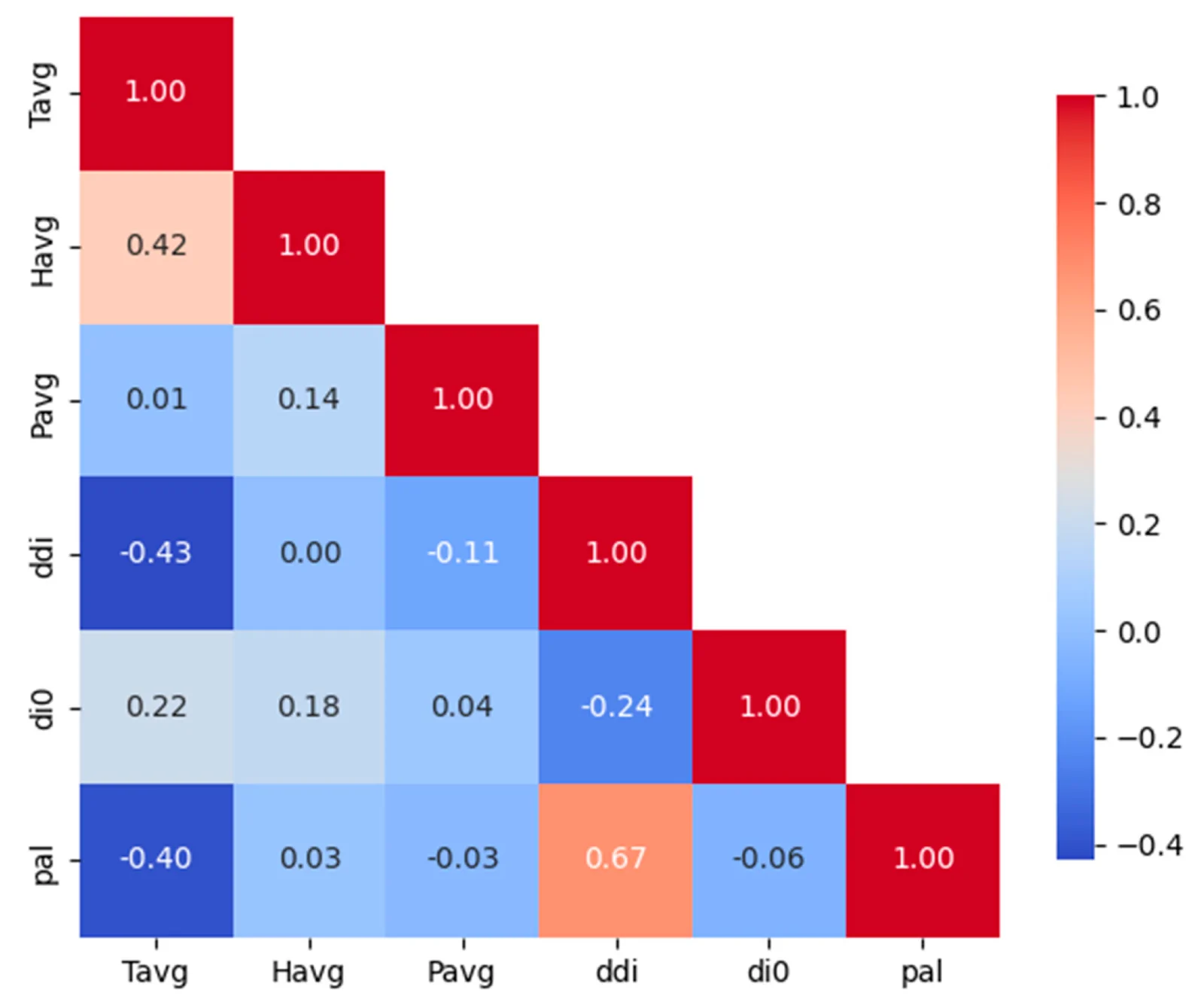
Pearson correlation matrix of key environmental and particulate variables. Tavg represents daily average temperature, Havg represents daily average humidity, Pavg represents daily average particulate concentration, ddi, di0, and *Pal* represent the daily change rate of disease index, the initial disease index on the day, and the pathogen (*Pseudomonas amygdali* pv. *lachrymans*, *Pal*) concentration, respectively.

The results of the correlation matrix indicate that temperature and humidity exhibit a certain degree of coherence, suggesting that both variables fluctuate in a coordinated fashion on a daily scale (Figure 1). In contrast, the average particulate concentration shows generally weak correlations with temperature and humidity, implying that particulate variations are largely independent of the other factors. The rate of change in disease index is strongly positively correlated with pathogen aerosol concentration, indicating that pathogen aerosols are a primary driver of disease progression. Moreover, low temperatures promote aerosol release and accelerate disease development.

**Figure 2:**
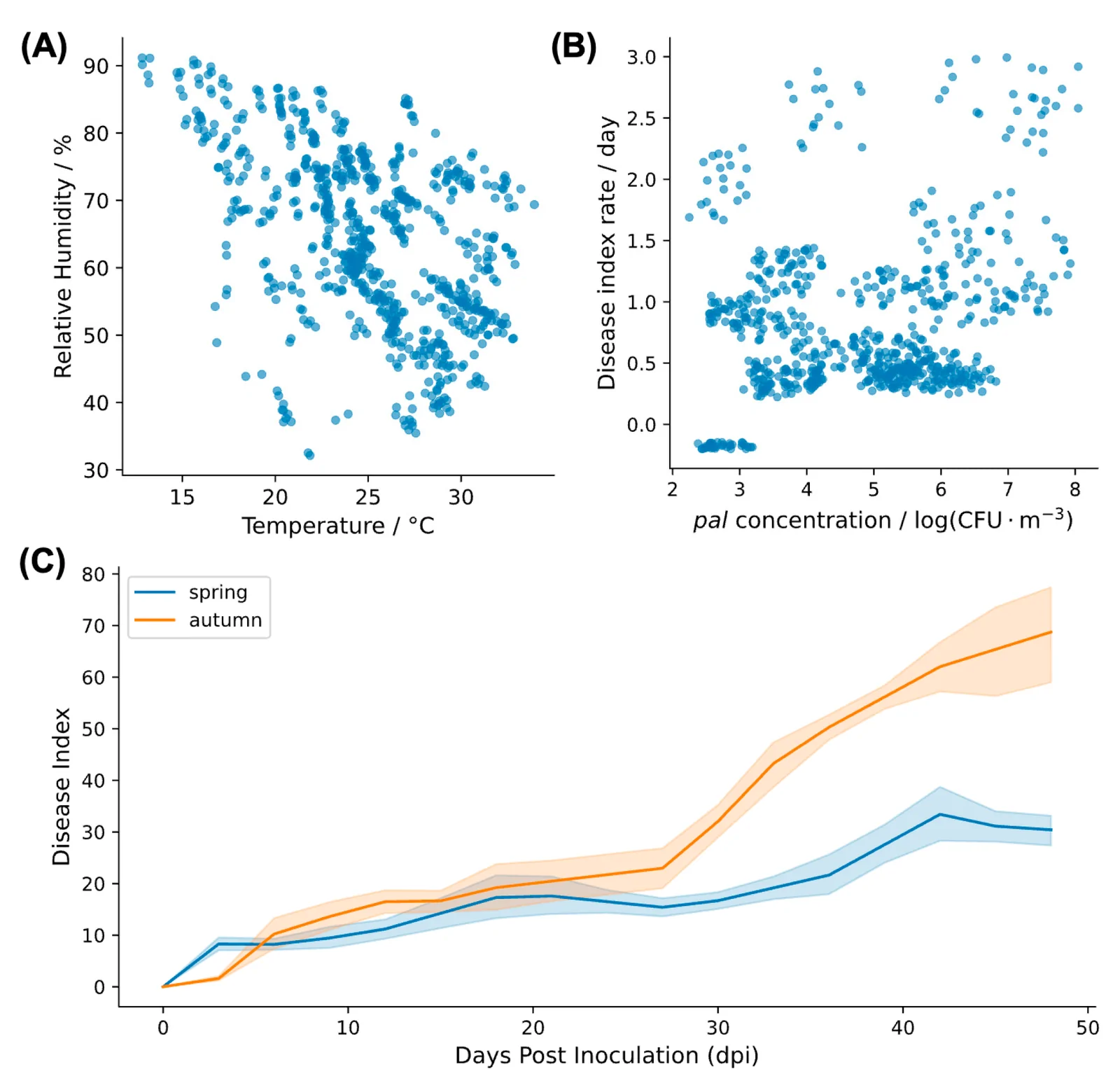
Feature distributions in the dataset. (**A**) Combined distribution of average temperature and humidity; (**B**) relationship between the change rate of disease index and pathogen concentration; (**C**) changes in disease index following days post-inoculation in spring and autumn experiments.

In spring and autumn inoculation experiments, disease initially spread rapidly in both seasons. However, seasonal differences resulted in significant variations in final disease severity (Figure 2). In autumn, relatively favorable environmental conditions, larger diurnal fluctuations in temperature and humidity, and stronger greenhouse airflow facilitated pathogen aerosol dispersion and rapid disease progression, leading to higher final disease index values. In contrast, following spring inoculation and the subsequent transition into summer, cucumber plants produced large numbers of new leaves, and the elevated temperatures constrained pathogen multiplication and dispersal. As a result, the plant-level disease index tended to reach a stable level and even showed a slight decrease during certain periods.

### 2.2. Pathogen Aerosol Surveillance Model

The pathogen aerosol monitoring model constructed from spring and autumn data demonstrated stable predictive performance on the validation set, with nonlinear tree-based models outperforming linear models. The Extra Trees (ET) model achieved the best and most robust performance across five independent iterations, with a mean coefficient of determination (R^2^) of 0.884 (±0.007), a root mean square error (RMSE) of 0.454 (±0.072), a mean absolute error (MAE) of 0.332 (±0.020), and a mean absolute percentage error (MAPE) of only 7.64% (±0.29%), indicating that the model can accurately and consistently estimate airborne pathogen concentrations from particulate and environmental sensor data. The Random Forest (RF) model performed slightly worse, with an average R^2^ of 0.828 (±0.010) and slightly higher error metrics but still maintained good generalization and high stability. Extreme Gradient Boosting (XGBoost) achieved an average R^2^ of 0.797 (±0.022) with somewhat larger prediction errors and wider confidence intervals, possibly due to its higher parameter sensitivity on small-to-medium datasets. The Decision Tree (DT) and Linear Regression (LR) models, used as baselines, showed poor and highly variable performance (average LR R^2^ = 0.345 ± 0.114; DT R^2^ = 0.620 ± 0.087), demonstrating that pathogen particle release is nonlinear and cannot be effectively captured by linear relationships with temperature, humidity, vapor pressure deficit (VPD), and particulate peak behavior.

The results in Table 1 confirm that tree-based ensemble methods (particularly ET and RF) effectively and robustly handle the nonlinear structure of the monitoring data. Considering the robust model performance metrics across multiple runs, the monitoring framework developed here, based on low-cost particulate sensors and environmental factor data, can provide continuous estimates of pathogen aerosol concentrations without requiring biologically specific detection.

**Figure 3:**
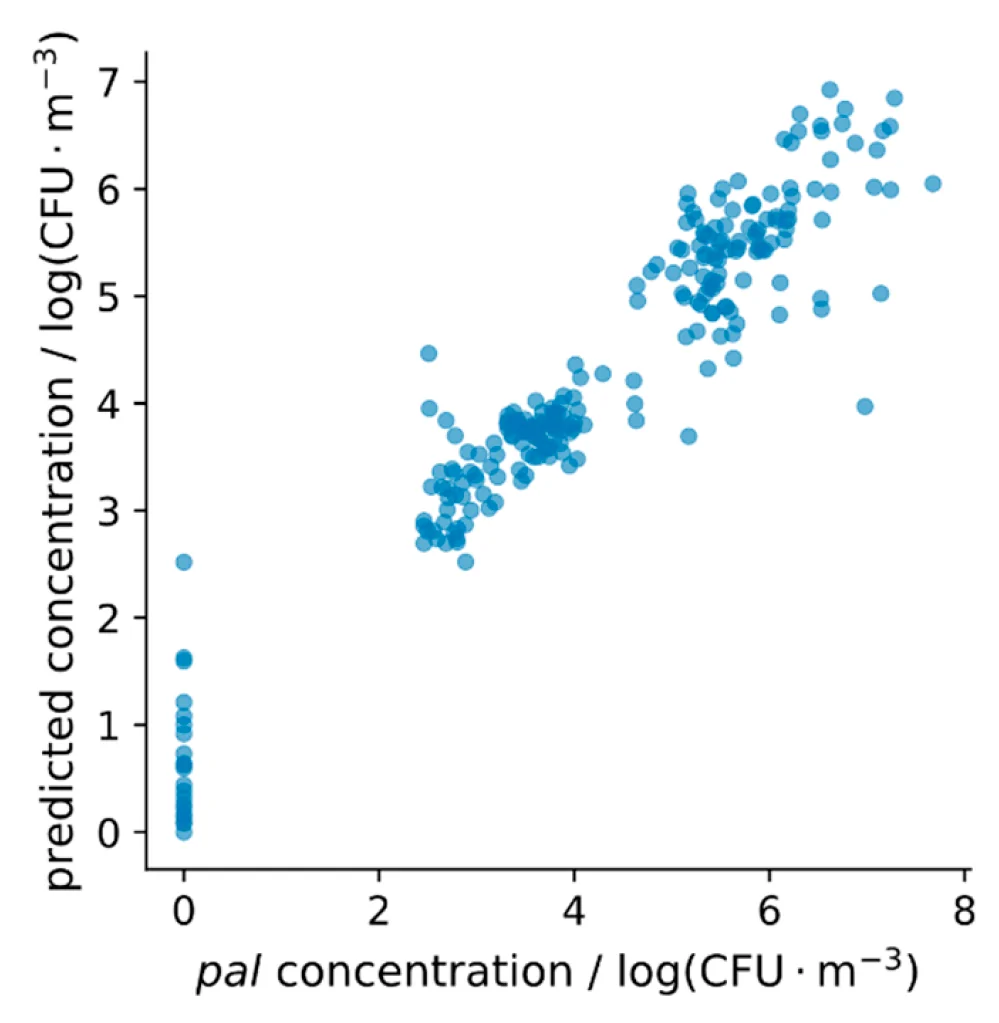
Predicted pathogen aerosol concentrations based on particulate matter sensors, temperature and humidity data. For healthy cucumber plants, the *Pal* (*Pseudomonas amygdali* pv. *lachrymans*) concentration is zero, yet the model generates non-zero predictions, most of which remain below the pathogenic threshold.

The pathogen aerosol surveillance model developed from particulate matter and environmental sensor data demonstrated strong performance in predicting the logarithmic concentration of airborne pathogens, achieving a MAE of 0.332. This level of accuracy indicates that the model reliably captures the dynamics of pathogen aerosols. For samples collected from healthy plants, predictions were not strictly zero due to fluctuations in background particulate matter within the greenhouse; however, the predicted values remained consistently low (Figure 3). Based on previous aerosol transmission studies and our preliminary greenhouse inoculation trials, an operational early-warning threshold of approximately 200 CFU m^−3^ was selected [13], corresponding to a logarithmic value of about 2.3. This value was not regarded as an absolute infection boundary, but was used because previous studies indicated that *Pal* aerosol concentrations near this level could initiate infection under favorable greenhouse conditions, and our preliminary trials showed that the daily disease index change rate began to increase more clearly when airborne concentrations approached or exceeded this range. In the present dataset, 95.24% of healthy-plant samples had predicted values below this threshold.

**Figure 4:**
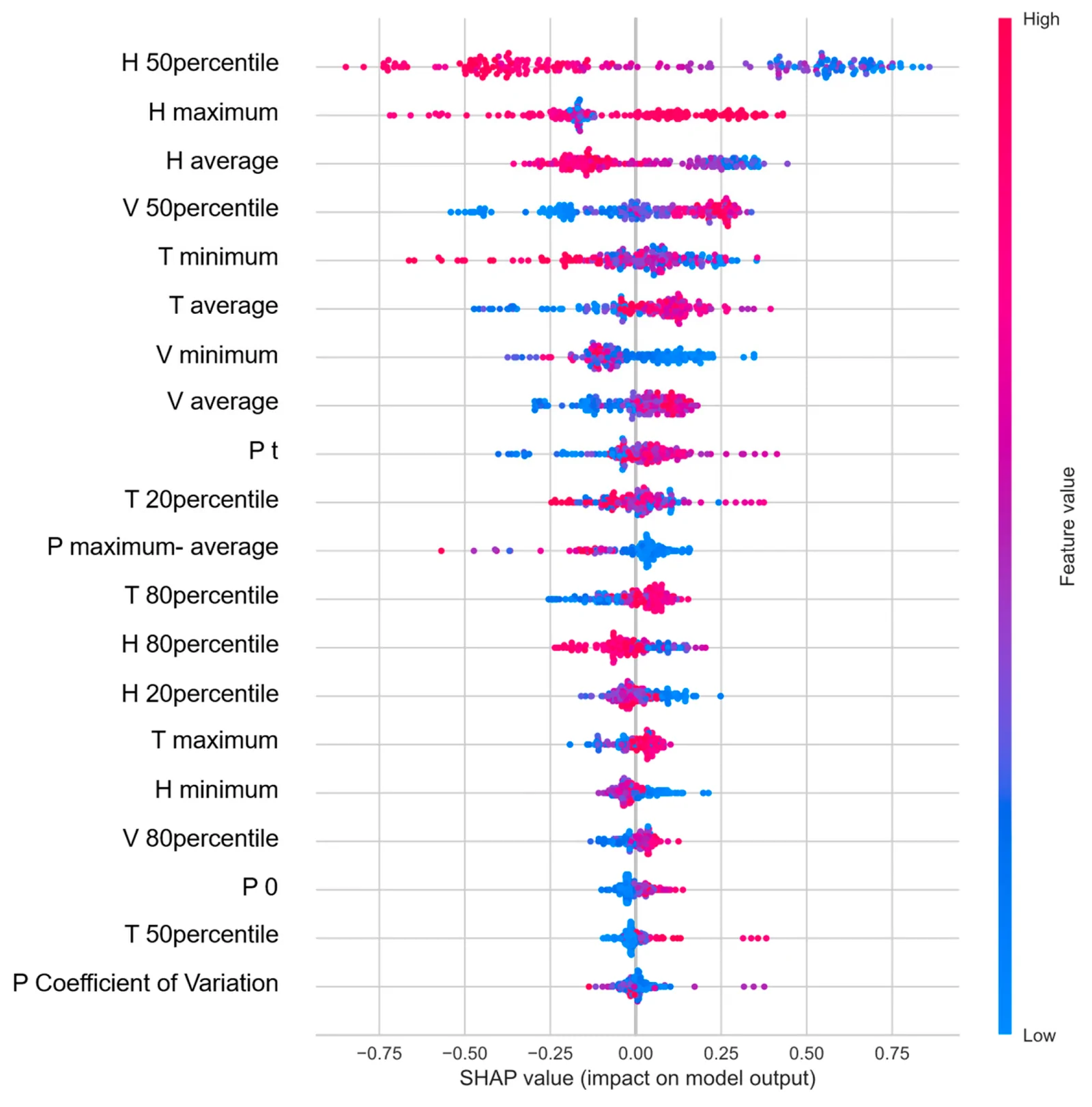
SHapley Additive exPlanations (SHAP) summary plot of the monitoring model. Labels indicate feature names and feature-engineering methods. “T H P V” represents temperature, humidity, particle variables and vapor pressure deficit (VPD). The additional “Pt” represents the maximum particulate concentration at 0:00 or 15:00, and “P0” represents the maximum particulate concentration at 0:00.

SHapley Additive exPlanations (SHAP) interpretability analysis further reveals the model’s logic in predicting airborne pathogen aerosol concentrations and quantifies the contribution of different input features to model outputs (Figure 4). In the monitoring model, environmental humidity and VPD are key influencing factors with high average contributions, indicating that air humidity plays a decisive role in pathogen release and accumulation. Lower humidity promotes leaf transpiration and gas exchange, conditions that favor the escape of *Pal* from leaf surfaces into the air as aerosol, which is reflected in the SHAP plot as a strong positive contribution increasing predicted values. In addition, the daily range of humidity shows high Shapley values, suggesting that short-term humidity accumulation or sudden rises may be closely associated with instant aerosol release events. The positive contributions of the 20th and 80th percentiles of temperature in the SHAP plot indicate that the proportion of high- and low-temperature periods jointly determines the time for pathogen release. In particular, higher minimum temperature percentiles usually correspond to sustained high-temperature conditions, under which *Pal* survival and airborne retention are reduced, with SHAP scatter points concentrated in regions lowering predicted values. In contrast, the mean temperature contributes less, indicating that a single temperature level cannot capture the complexity of pathogen release, whereas intraday temperature sequence better reflects the underlying biological mechanisms.

Among particulate features, the peak deviation features constructed from the difference between the maximum particulate concentrations at 0:00 or 15:00 and the daily average show prominent contributions. Their SHAP values exhibit strong positive effects at high feature values.

### 2.3. Results of Disease Index Prediction Model

In the prediction model for the daily rate of change in the disease index constructed from pathogen aerosol concentration and environmental features, several nonlinear regression algorithms show strong predictive performance (Table 2). While ET was the optimal algorithm for the surveillance task (Section 2.2), the XGBoost model reached the best practical performance for this specific forecasting task. Although the mean R^2^ of XGBoost (0.874 ± 0.028) was comparable to that of ET (0.897 ± 0.020), XGBoost exhibited a notably lower mean absolute percentage error (MAPE) (15.35% vs. 19.97%). When predicting the relative trend of disease expansion (i.e., the disease index change rate), minimizing percentage errors is of greater value for practical agricultural early warning. Therefore, XGBoost was selected as the primary forecasting model. It achieved an average R^2^ of 0.874 (±0.028) on the validation set, with an RMSE of 0.191 (±0.021), an MAE of 0.095 (±0.006), and a MAPE of 15.35% (±1.98%), indicating that it captures daily-scale disease progression dynamics with good accuracy and stability. LR model performs the worst, with an average R^2^ of 0.364 (±0.040) and a MAPE of 73.18% (±3.13%), showing that changes in the disease index are jointly driven by pathogen concentration, temperature and humidity conditions and their fluctuation patterns, and cannot be adequately represented by a linear model.

**Figure 5:**
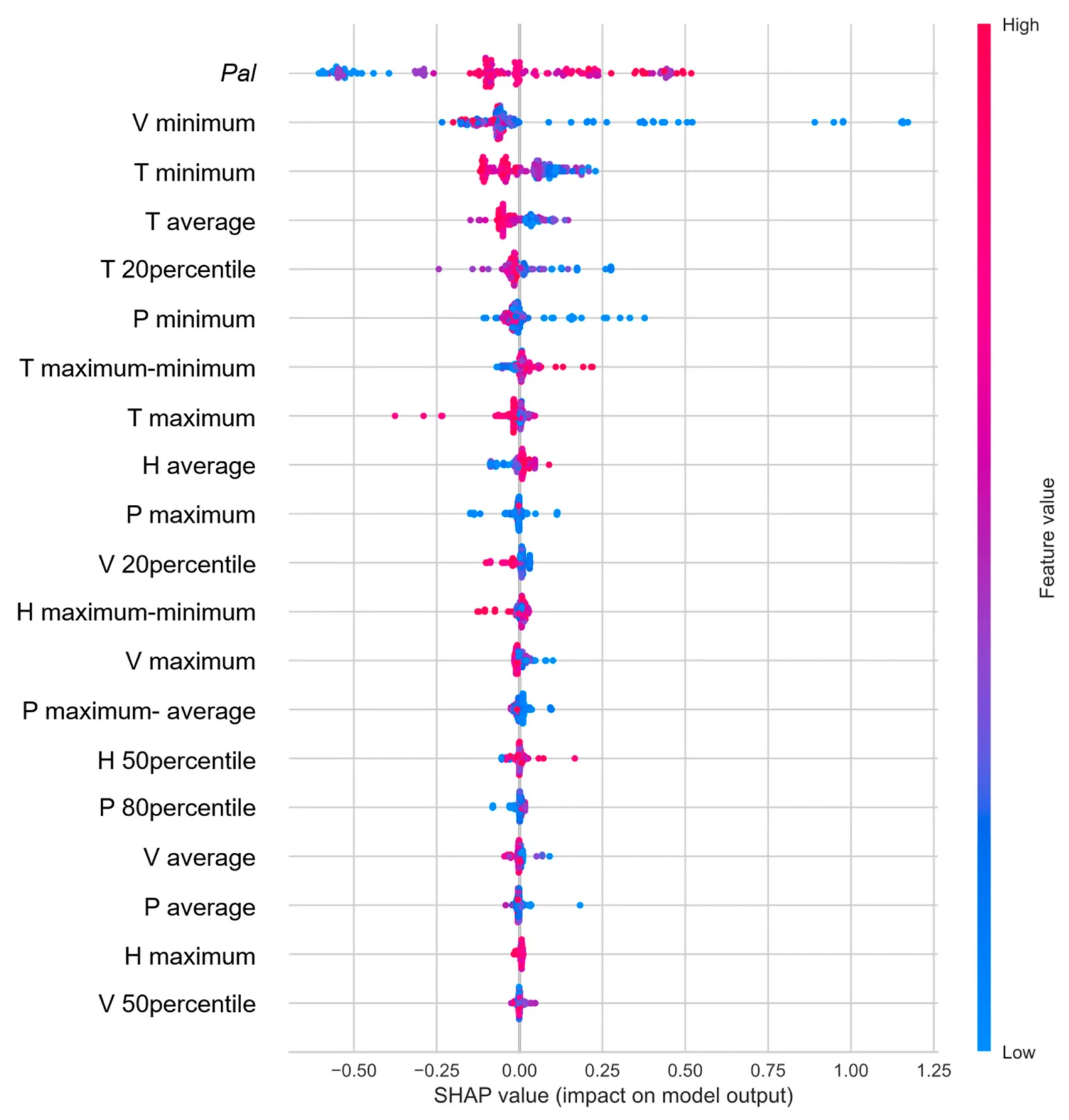
SHapley Additive exPlanations (SHAP) summary plot of the prediction model. Labels indicate feature names and feature engineering methods. “T H P V” represents temperature, humidity, particle variables and vapor pressure deficit (VPD), and the additional “*Pal*” label represents the measured concentration of the pathogen (*Pseudomonas amygdali* pv. *lachrymans)*.

SHAP feature contribution analysis reveals how the model makes decisions when predicting the rate of change in the disease index and quantitatively shows the relative importance of environmental factors and pathogen aerosol indicators during disease development (Figure 5). Airborne pathogen aerosol concentration is the central driver of disease index variation, and its Shapley values show a strong positive effect under high concentrations. This indicates that when pathogen load in the air increases, the model tends to predict faster disease expansion even if environmental conditions are not fully optimal.

VPD also exhibits a strong contribution in the prediction model. Low VPD samples fall in positive contribution ranges and increase the predicted rate of change in the disease index. High humidity conditions corresponding to low VPD support pathogen survival and attachment on leaf surfaces, extend the persistence of water films, and promote pathogen movement across leaf tissues, thereby enhancing infection efficiency.

The temperature features show a clear pattern in their contributions to the model. The 20th percentile of temperature has a relatively strong impact, and higher values act to suppress disease expansion, indicating that sustained high temperatures reduce pathogen activity and slow disease progression. In contrast, the mean and maximum temperature contribute less, suggesting that disease development is driven more by intraday temperature dynamics than by absolute temperature levels. For example, the 20th percentile represents the temperature exceeded for 80% of the day; a higher value reflects prolonged high-temperature conditions, which inhibit disease expansion. Under such sustained high temperatures, the model predicts a marked reduction in disease progression speed. The fluctuation patterns of particulate concentration, including extremes and percentiles, indicate that variations in particle dynamics are linked to pathogen release processes and resuspension events driven by local air movement.

## 3. Discussion

Accurate prediction of airborne disease epidemics requires models capable of representing complex interactions among pathogen load, environmental conditions, and host disease development. In this study, the pathogen aerosol surveillance model and the disease index prediction model both showed that nonlinear algorithms performed better than linear regression, indicating that the release, accumulation, and infection processes of airborne *Pal* are not governed by simple linear responses to temperature, humidity, or particulate matter alone [38]. Tree-based ensemble models, particularly ET and XGBoost, effectively captured cumulative environmental effects, threshold responses, and the coupling between particulate dynamics and pathogen release. The strong performance of these algorithms is consistent with their advantages in modelling nonlinear feature interactions and heterogeneous tabular data [39,40]. In comparison with representative plant disease forecasting studies that mainly used meteorological or environmental variables and reported R^2^ values generally in the range of approximately 0.70–0.80 [26,28], the ET-based surveillance model in this study achieved a mean R^2^ of 0.884. This comparison should be interpreted cautiously because crop species, pathogens, datasets, and prediction targets differed among studies. Nevertheless, the narrow 95% confidence intervals obtained from five independent modeling iterations, especially for R^2^ (0.884 ± 0.007) and MAPE (7.64% ± 0.29%), indicate that the performance was stable within the present dataset and support the added value of incorporating PM-derived aerosol fluctuation features with microclimate variables. This improvement suggests that real-time particulate matter signals provide valuable physical information related to airborne pathogen dynamics and can enhance disease early warning when combined with environmental sensing.

A major advantage of the proposed framework is that it introduces pathogen-related information into the early-warning process. Conventional disease forecasting approaches usually evaluate whether environmental conditions are favorable for disease occurrence, but they rarely quantify whether sufficient pathogen inoculum is present. However, even under suitable temperature and humidity conditions, disease development cannot occur without adequate pathogen load, which is consistent with the disease triangle concept emphasizing the joint requirement of a susceptible host, a virulent pathogen, and a favorable environment [32,41]. In this study, low-cost particulate matter sensors were used to continuously estimate airborne pathogen concentrations, while qPCR-based pathogen quantification was triggered only when the predicted concentration exceeded the pathogenic threshold. This risk-triggered workflow can reduce unnecessary manual sampling and laboratory detection while retaining the accuracy of molecular confirmation at key decision points. The finding that 95.24% of healthy-plant samples were predicted below the pathogenic threshold further indicates that the model can effectively identify low-risk conditions and support routine greenhouse disease surveillance.

The SHAP analysis further clarified the biological basis of the model predictions. SHAP has been widely used to interpret complex machine learning models by quantifying the contribution of individual features to model outputs [42,43]. In this study, airborne pathogen concentration was the dominant factor driving the rate of change in the disease index, confirming that pathogen load directly constrains the speed of disease expansion. VPD also showed a strong contribution to disease development. Low VPD, which corresponds to high air moisture and low evaporative demand, can prolong the persistence of water films on leaf surfaces, promote bacterial survival and movement, and enhance infection efficiency. Leaf wetness and high humidity are widely recognized as critical factors affecting the infection process of many foliar pathogens [44,45]. This result is also consistent with previous findings that high humidity favors pathogen aerosol survival, deposition, and infection in greenhouse environments [4,46]. Compared with relative humidity alone, VPD integrates the effects of temperature and atmospheric moisture demand, making it a more sensitive indicator for describing the microclimatic conditions associated with bacterial disease progression.

Temperature dynamics also played an important role in regulating disease development. The contribution of temperature percentile features was greater than that of mean temperature, indicating that intraday temperature distribution better reflects pathogen activity than a single daily average. In particular, sustained high temperatures, represented by high values of the 20th temperature percentile, were associated with reduced disease progression. Temperature is known to affect bacterial survival during aerosolization, pathogen viability, and infection efficiency [14,15,16]. This pattern provides biological support for the observed plateau or slight decline in disease index during the later stage of the spring trial, when the experiment gradually entered warmer summer conditions. However, this apparent decline did not indicate true disease recovery, because lesions on previously infected leaves continued to enlarge. Instead, the rapid production of new cucumber leaves diluted the plant-level disease index, suggesting that disease progression is jointly affected by pathogen infection, seasonal microclimate, and host growth status. Therefore, future disease prediction models could be improved by integrating greenhouse crop growth models with pathogen and environmental monitoring data.

Although low-cost particulate matter sensors cannot distinguish biological particles from inorganic dust or other aerosol sources, their dynamic signals still contribute to pathogen surveillance and disease prediction. It must be noted that PM concentrations alone cannot reliably reflect the abundance of *Pseudomonas amygdali*, as they are easily influenced by irrigation, ventilation, and human activity. However, in this framework, PM data are never evaluated in isolation. By integrating PM signals with specific microclimate variables (such as VPD, temperature, and humidity percentiles), the machine learning models learn the distinct multidimensional “signatures” of pathogen release events, filtering out noise from routine greenhouse activities. Particulate peak features at key release periods, especially around 15:00 and 00:00, were important in the monitoring model, indicating that particulate fluctuations can partially capture aerosol release or resuspension events related to pathogen dispersal. This is consistent with previous studies showing that airborne pathogen particles in greenhouses exhibit distinct diurnal release patterns regulated by humidity and airflow conditions [5,38]. When these signals were interpreted together with humidity, VPD, and temperature features, the model was able to compensate to some extent for the lack of biological specificity in the sensors. This provides a practical compromise between monitoring cost and biological relevance, especially for long-term deployment in commercial greenhouses. Nevertheless, future improvements could incorporate bioaerosol sensors based on biological fluorescence, such as ultraviolet-induced fluorescence, which has been used for real-time detection and classification of biological aerosol particles [47,48], to distinguish biological particles more directly and further improve monitoring accuracy.

The proposed framework also has practical implications for greenhouse disease management. Because the model links predicted disease risk with controllable environmental variables, it can provide guidance for interventions such as reducing prolonged high-humidity periods, improving ventilation, and limiting aerosol accumulation. In this sense, the model is not only a predictive tool but also a decision-support system that connects pathogen monitoring with management actions. However, several limitations should be considered. DNA-based qPCR cannot distinguish viable bacterial cells from DNA released by dead cells, which may overestimate actual infection risk. Future studies should consider RNA-based detection, viability qPCR, or culture-based methods to better quantify viable pathogen loads. The present framework was tested in enclosed greenhouse conditions, where airflow and background aerosol sources are relatively constrained. Its direct application to open-field environments, where wind dynamics and non-biological particles are more complex, would require further calibration and validation. Although the feature-engineering-based machine learning approach achieved high predictive accuracy, future work could incorporate forecasted environmental variables to enable forward-looking disease risk prediction before unfavorable conditions occur.

## 4. Materials and Methods

### 4.1. Integrated Early-Warning System Architecture

**Figure 6:**
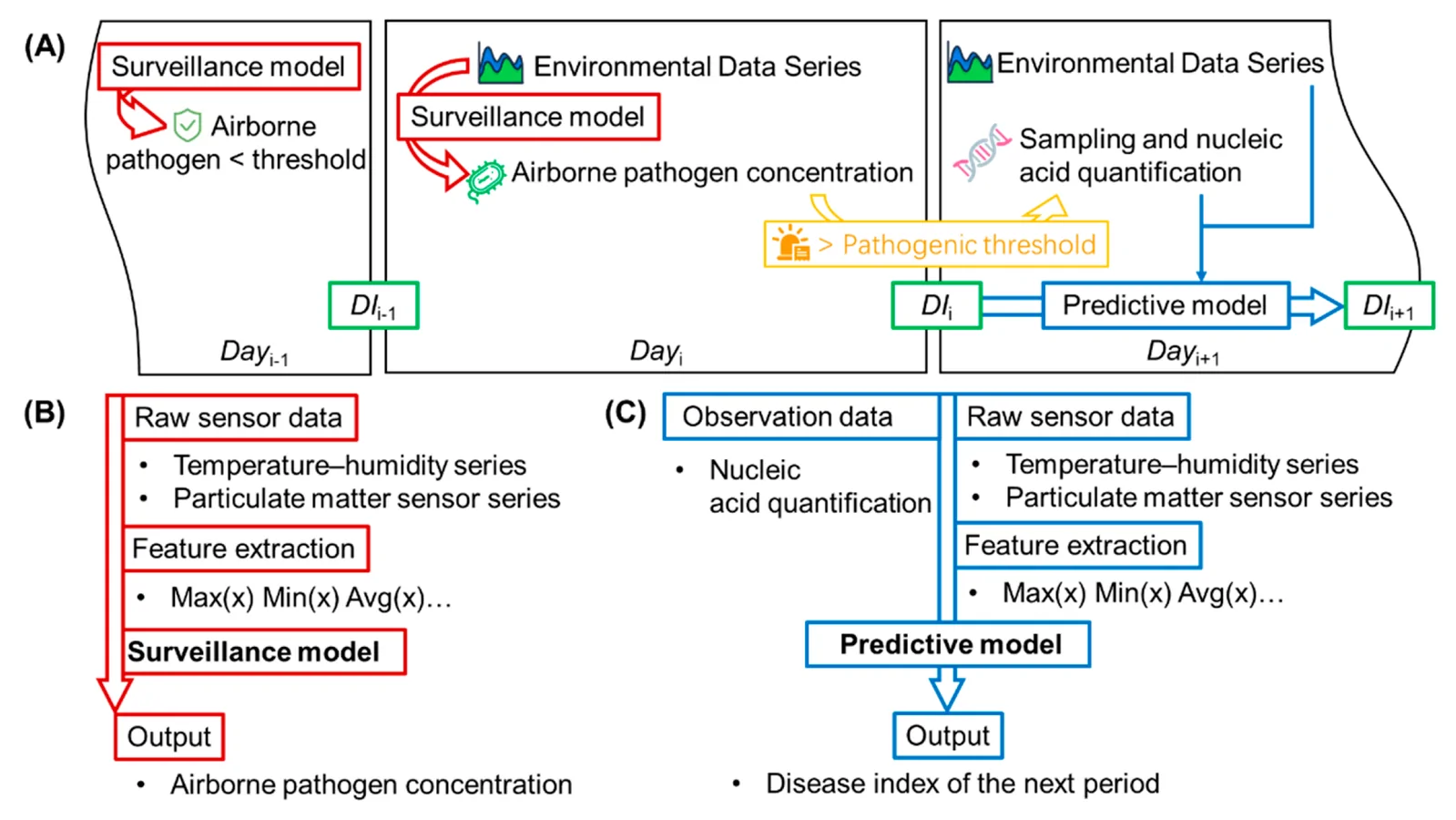
Early-warning workflow for cucumber angular leaf spot. (**A**) During routine monitoring, the surveillance model estimates the concentration of pathogens via sensors. When the predicted concentration exceeds the threshold, nucleic acid detection is performed, and a prediction model is used to forecast changes in the disease index. (**B**) In daily monitoring, raw sensor observations are processed through feature extraction and input into the surveillance model to obtain predicted pathogen concentrations. (**C**) The prediction model takes nucleic acid results and environmental sensor data as inputs and outputs the rate of change in the disease index. Red elements denote the surveillance model workflow, blue elements represent the predictive model workflow, green boxes indicate the disease index (DI), and yellow highlights the pathogenic threshold trigger.

An integrated early-warning workflow for cucumber bacterial angular leaf spot was developed, comprising three main components: environmental sensing, pathogen surveillance and early warning, and disease prediction (Figure 6). The hardware of the developed system integrates a central microcontroller with specific environmental and particulate sensors, communicating via a wireless module to process and transmit data. Specifically, the system continuously collects environmental data such as temperature, humidity, and particulate matter through sensors deployed within the greenhouse. A surveillance model then estimates the concentration of airborne pathogens. When the predicted concentration exceeds a predetermined pathogenic threshold established from preliminary experiments (approximately 200 CFU m^−3^, corresponding to a logarithmic value of 2.3, where the daily rate of disease index change was observed to accelerate significantly) [13], greenhouse managers are notified to conduct air sampling and quantitative nucleic acid detection to verify pathogen load. The quantification results are then combined with environmental data and input into the prediction model to calculate the trend of disease index changes for the next monitoring cycle.

### 4.2. Sensor Design and Data Acquisition

**Figure 7:**
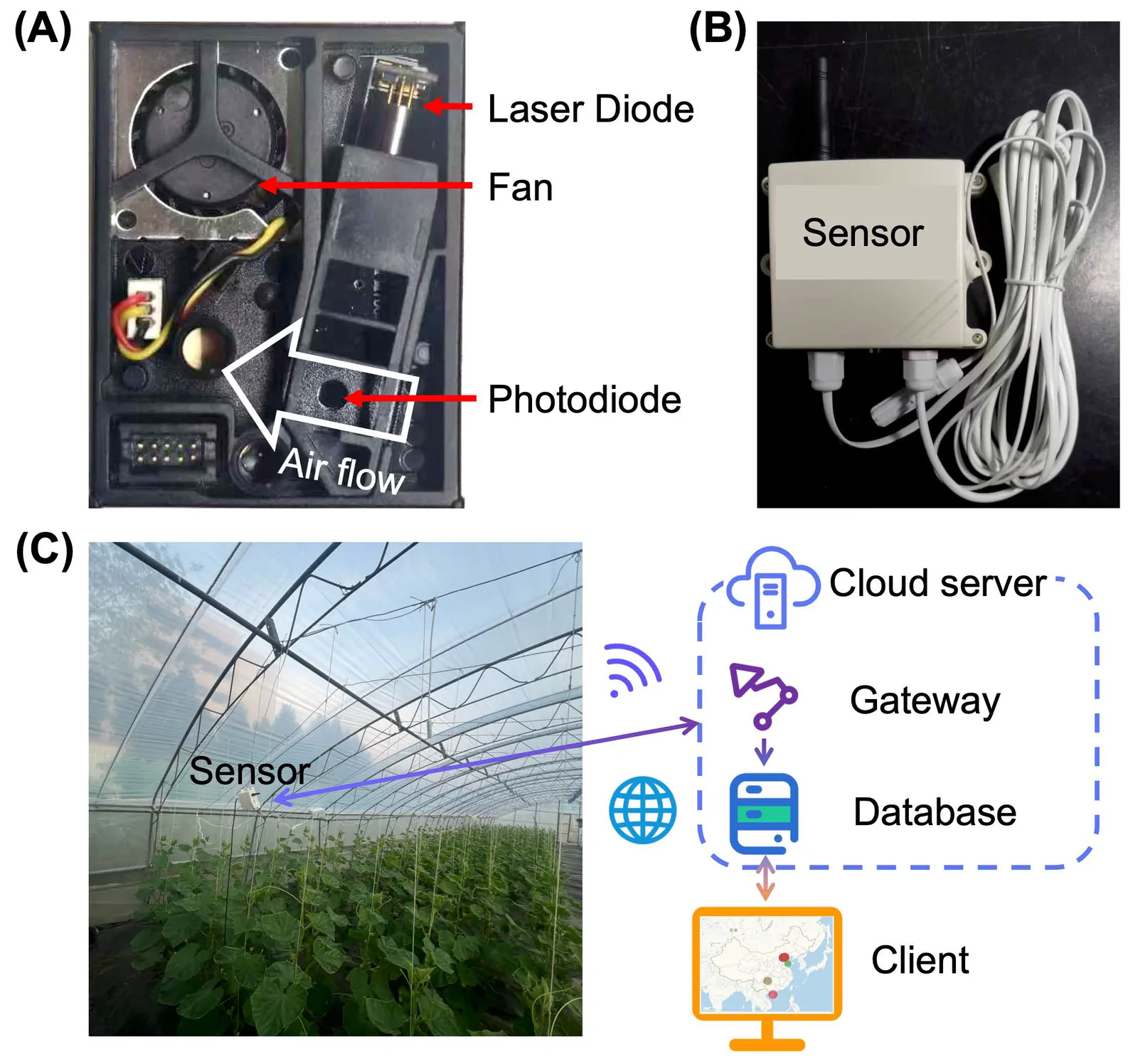
The environmental data acquisition workflow uses IoT (Internet of Things) sensors. (**A**) Internal structure of the particulate matter sensor, which measures particle concentrations by capturing the scattered laser signal with a photodiode; (**B**) Environmental sensor, equipped with an external temperature and humidity sensor; (**C**) The sensor transmits data to the server via a 4G network, where the data are stored in a database.

To achieve continuous remote monitoring and early warning of airborne pathogens in greenhouses, a pathogen monitoring sensor system was developed. A high-precision temperature and humidity sensor (SHT40, Sensirion, Switzerland, Stäfa, Switzerland) was installed externally, with its probe positioned near the plant canopy to closely represent the environmental conditions relevant to pathogen infection and dissemination (Figure 7A). Since cucumber bacterial angular leaf spot can be transmitted via aerosols, a particulate matter sensor (PMS7001, Plantower, Beijing, China; a legacy model purchased for this study, which has specifications equivalent to the current PMS7003 model) was incorporated to measure spatial particle concentrations. Its detection range (0.3–10 μm) covers the main size distribution of bacteria- and pathogen-associated aerosol particles, enabling real-time monitoring of airborne pathogen aerosols and dust particles [13,14]. The sensor system, integrated with data acquisition and a 4G wireless module, is controlled by an STM32G070 microcontroller for raw data collection and preliminary processing. Temperature and humidity, which significantly influence the disease’s spread, are continuously monitored alongside aerosol particle concentrations (Figure 7B). The collected data are transmitted in real time to a remote cloud server via the 4G module. On the server, a time-series database is used for data storage and management, and a disease early-warning algorithm based on the sensor data is deployed (Figure 7C). The server integrates a visualisation platform to display dynamic trends in temperature, humidity, particulate matter, and model predictions, providing a data foundation for continuous monitoring and decision support.

### 4.3. Experimental Greenhouse and Trial Design

To ensure spatial independence and validate the system’s robustness across different microclimates and greenhouse structures, field inoculation experiments were conducted in three distinct greenhouses located in the Haidian and Pinggu Districts of Beijing, China (Table 3). The experimental facilities included a plastic arch tunnel covered with a single layer of polyethylene film (Greenhouse I) and two solar greenhouses (Greenhouses II and III). All greenhouses were equipped with top and side roll-up ventilation systems, operated manually daily to regulate internal temperature and humidity.

For the spring trial, seedlings were transplanted on 24 April 2025 into Greenhouses I and II. For the autumn trial, seedlings were transplanted on 20 August 2025 into Greenhouses I and III. Prior to transplanting, cucumber plants were cultivated in a seedling chamber until the first true leaf was fully expanded. Within each greenhouse, a wide-narrow row planting pattern was used (plant spacing: 25 cm; wide/narrow row spacing: 70 cm/50 cm) to ensure uniform ventilation and light conditions.

**Figure 8:**
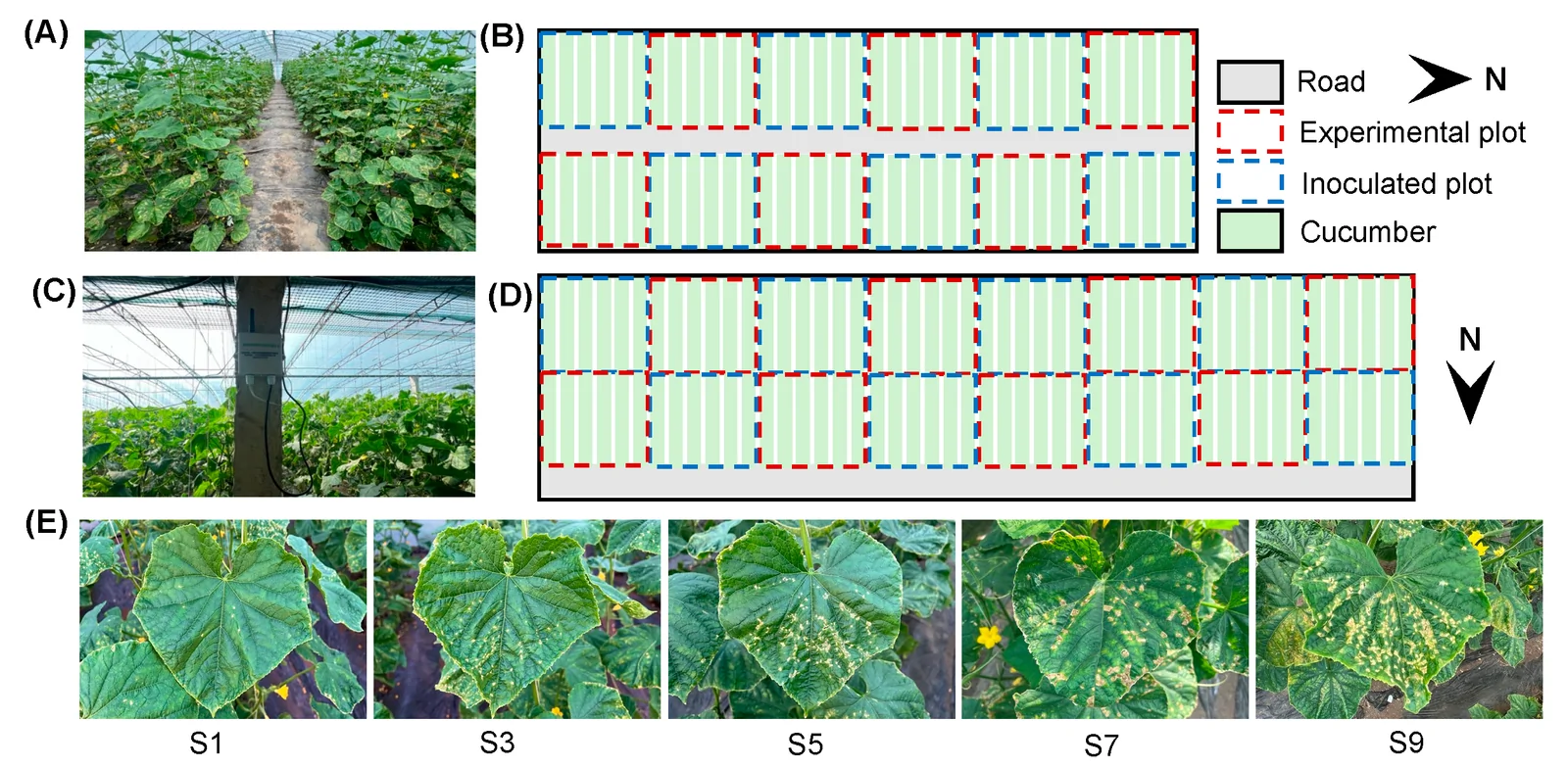
Layout of the greenhouse experimental plots and severity grading of cucumber angular leaf spot. (**A**) Field occurrence of cucumber angular leaf spot in the plastic arch tunnel (Greenhouse I). (**B**) Plot layout in the plastic arch tunnel, comprising six experimental plots and six inoculation plots arranged alternately. (**C**) Field occurrence in the solar greenhouses (Greenhouses II and III). (**D**) Plot layout in the solar greenhouses, comprising eight experimental plots and eight inoculation plots arranged alternately. (**E**) Representative cucumber leaves exhibiting different disease severity grades. Sensors and air samplers were deployed at the centre of each experimental plot, level with the plant canopy (approx. 1.5 m above ground).

Each season comprised a total of 14 experimental plots with uniform planting density. To simulate the natural dispersal of the pathogen, experimental plots and inoculation plots were arranged alternately. Specifically, the plastic arch tunnel (Greenhouse I) was divided into 12 blocks, comprising six experimental plots alternating with six inoculation plots (Figure 8A,B). The solar greenhouses (Greenhouses II and III) were divided into 16 blocks, comprising eight experimental plots alternating with eight inoculation plots (Figure 8C,D). Artificial inoculation was performed exclusively in the inoculation plots, and monitoring data were collected solely from the adjacent experimental (non-inoculated) plots.

Since the experiments were conducted in enclosed greenhouses, natural prevailing winds were restricted, and internal airflow was primarily driven by thermal convection and routine side-ventilation. Fourteen sensor nodes were deployed in total per season (one per experimental plot). These sensors were positioned at the centre of each plot at canopy height (approximately 1.5 m above the ground) (Figure 8E).

### 4.4. Disease Index Assessment

Disease indices were conducted every day in the survey plot. In each plot, 10 cucumber plants were randomly selected, and the leaves were assessed for disease index. Diseased leaves were graded according to the percentage of leaf area covered by lesions: S0 = no lesions; S1 = 0–5%; S3 = 5–25%; S5 = 25–50%; S7 = 50–75%; S9 = 75–100%. The disease index was calculated based on the number of diseased leaves at each severity level, using the following formula:

(1) $$DI=\frac{\sum \left(n\times v\right)}{N\times V}\times 100$$

where DI is the disease index, *n* is the number of diseased leaves at each severity level, *v* is the corresponding severity grade value, *N* is the total number of leaves investigated, and *V* is the highest severity grade value (9 in this study).

### 4.5. Aerosol Sampling and qPCR Quantification

Once daily, following each disease index survey, aerosol samples were collected. Based on previous studies, the daytime peak of pathogen release occurs at 15:00 daily [38], so aerosol sampling was conducted at 15:00. The daily change in the disease index was calculated to establish a model linking environmental data and aerosol concentrations with the rate of disease development. Aerosol samples were collected using a portable high-flow bioaerosol sampler (WL-4H, Beijing Gongjiang Bio, Beijing, China). During sampling, airborne aerosols were captured in sterile centrifuge tubes containing 2 mL of 0.1% (*v*/*v*) Tween-20 solution. The sampling flow rate was set at 40 L·min^−1^ for a duration of 2 min. Immediately after sampling, the tubes were sealed and transported to the laboratory. The solution was briefly vortexed to homogenise. Since *Pal* is a Gram-negative bacterium, a thermal lysis method was employed [6]. The solution was heated at 95–100 °C for 5 min and centrifuged, and the supernatant was directly used as the template for subsequent quantitative detection. Pathogen concentrations at the time of sampling were calculated from the established standard curve. The standard curve was generated using a 10-fold serial dilution of a known concentration of *Pal* plasmid DNA (ranging from 10^3^ to 10^8^ copies μL^−1^). The curve exhibited an R^2^ of 0.995 and an amplification efficiency of 98.4%.

Quantification of the pathogen was performed using real-time quantitative PCR (qPCR) on an ABI 7500 Real-Time PCR System (Applied Biosystems, Foster City, CA, USA). The primers used were Pslgap1-F (5′-TCGGCGACGCAATCAAT-3′) and Pslgap1-R (5′-GGTGGTTTCACGCTTCAGG-3′) [13]. The qPCR reaction mixture had a total volume of 20 μL, comprising 10 μL SYBR qPCR Master Mix (Novizan, Beijing, China), 0.5 μL each of forward and reverse primers (final concentration 200 nM), and 9 μL of DNA template. The reaction conditions were as follows: an initial denaturation at 95 °C for 15 min, followed by 39 cycles of 95 °C for 15 s and 62 °C for 30 s. After amplification, a melting curve analysis was performed from 60 °C to 90 °C with a ramp rate of 0.5 °C 30 s^−1^. Each sample was analysed in triplicate, and both the amplification and melting curves were examined to verify the specificity and accuracy of the detection. No-template controls (sterile water) and field blanks (open tubes without sampling flow) were included in every qPCR run to rule out contamination and confirm that detected DNA was derived from the sampled aerosols.

### 4.6. Feature Extraction and Processing

Temperature, humidity, and particulate matter data collected by the monitoring sensors over the entire experimental period (lasting approximately 8 weeks for each season) were recorded at 15 min intervals, with each day treated as a separate time series. During the experiment, sensors occasionally experienced unexpected downtime due to battery issues or weather conditions. To ensure data quality, only complete daily time series were retained, while incomplete sequences were discarded. Each day was treated as an individual analysis period, and statistical features were extracted from the raw observation sequences of each monitored factor to construct the model input feature set. For temperature and humidity data, the maximum, minimum, mean, 20th, 50th, and 80th percentiles, as well as the difference between the maximum and minimum values, were calculated to characterise the overall daily level and the range of diurnal fluctuations. Additionally, vapor pressure deficit (VPD) was calculated from the temperature and relative humidity data to reflect the influence of air moisture conditions on the microenvironment for disease spread [2,14]. The calculation formula for VPD is as follows:

(2) $$\mathrm{VPD}=e_{s}\left(T\right)\times \left(1-\frac{RH}{100\%}\right)\;$$

where $e_{s}(T)$ is the saturation vapor pressure (kPa) corresponding to temperature T, and *RH* is the relative humidity (%).

Previous studies on the diurnal dynamics of aerosol release indicated that peak release occurs at 15:00 and 00:00 [38]. For the particulate matter time series, in addition to extracting the same statistical metrics as for temperature and humidity, the timing of aerosol peaks relative to the daily mean was calculated to characterise the relative intensity and deviation of peak occurrences. Furthermore, to quantify the variability of particulate matter concentrations, the standard deviation and coefficient of variation were computed, reflecting the dynamic stability of aerosol concentrations throughout each day.

### 4.7. Construction of the Pathogen Aerosol Surveillance Model

To enable real-time monitoring of airborne pathogens causing cucumber angular leaf spot, a daily monitoring model for pathogen aerosols was developed using machine learning algorithms, including ET, RF, XGBoost, DT, and LR, with LR serving as a baseline to evaluate the advantages of nonlinear models in capturing dynamic features. For the LR model, multicollinearity was assessed using the Variance Inflation Factor (VIF < 5), and the normality of residuals was verified using the Shapiro–Wilk test. To integrate the different temporal resolutions, the 2 min qPCR pathogen concentrations were matched with the closest 15 min environmental sensor data point corresponding to the sampling time (15:00). All machine learning models and data preprocessing were implemented in Python (version 3.9) using the scikit-learn (version 1.0.2) and XGBoost (version 1.5.0) libraries. Hyperparameter optimization was performed using Grid Search coupled with 5-fold cross-validation. For the optimal ET surveillance model, the number of estimators was set to 200, and the maximum depth was set to None. For the best-performing XGBoost prediction model, the learning rate was 0.05, the maximum depth was 4, and the number of estimators was 150. The input data comprised sensor measurements collected on the day of sampling and corresponding pathogen concentrations determined by quantitative nucleic acid detection as the ground truth. To reduce scale differences, concentration data were log-transformed prior to model fitting.

### 4.8. SHAP-Based Model Interpretation

To further interpret the contribution of environmental factors and pathogen information to the model predictions, SHAP, a game-theoretic approach, was applied. Shapley values were calculated for each feature to quantify its average impact on the model output. Specifically, SHAP summary plots display the scatter distribution of Shapley values for each data point in the validation set, visualizing the positive or negative influence of input feature values on the predicted outcomes. Features were ranked by their mean absolute Shapley values to identify the most critical factors for disease prediction. SHAP analysis was implemented using the SHAP Python package (version 0.44.1, Lundberg, S., GitHub, Inc., San Francisco, CA, USA), providing a quantitative basis for interpretable analysis of environmental factors and pathogen dynamics.

### 4.9. Construction of the Disease Index Change Prediction Model

The presence and concentration of pathogens are the most critical factors in disease transmission and progression. The concentration of airborne pathogen aerosols directly determines the transmission rate of cucumber angular leaf spot, corresponding to the rate of change in the disease index. In addition, greenhouse temperature, humidity, and airflow conditions significantly affect pathogen dispersal efficiency and infection opportunities. Different temperature and humidity environments influence aerosol spread and pathogen viability, thereby promoting disease development. Therefore, integrating airborne pathogen concentrations with environmental factors provides a scientific basis for assessing disease risk.

The input features consisted of quantitative nucleic acid detection results and feature-engineered environmental sensor data (including the daily statistical metrics mentioned above and their one-day lag variables to capture delayed environmental effects), while the target variable was the rate of change in the disease index, quantifying disease progression to evaluate risk. The prediction model employed the same algorithms as the monitoring model. SHAP analysis was also used to assess the contribution of each feature to model predictions. Features were ranked by their mean Shapley values to identify the most critical environmental and pathogen-related factors, enabling dynamic prediction of disease index changes for the next monitoring period and providing precise early-warning support for greenhouse management.

### 4.10. Model Performance Evaluation

RMSE, MAE, MAPE, and R^2^ were selected to evaluate the predictive performance of both the monitoring and prediction models. For the monitoring model, the measured airborne pathogen concentrations served as the ground truth, and the accuracy of predictions based on environmental information was assessed. For the prediction model, the predicted rates of change in the disease index for each plot were used for evaluation. The formulas for these metrics are as follows:

(3) $$RMSE=\sqrt{\frac{\sum {\left(y_{i}-y_{i}^{\prime }\right)}^{2}}{n}}$$

(4) $$MAE=\frac{1}{n}\sum \left|y_{i}^{\prime }-y_{i}\right|$$

(5) $$MAPE=\frac{100\%}{n}\sum \left|\frac{y_{i}^{\prime }-y_{i}}{y_{i}}\right|$$

where $y_{i}$ is true values, $y_{i}^{'}$ is predicted values, n the total number of samples, $\bar{y}$ is the mean of n true values. For R^2^, values closer to 1 indicate better model performance, whereas lower values are preferable for the other metrics. To demonstrate the reliability and robustness of the model performance, the entire modeling process, including dataset splitting and model training, was independently repeated five times using different random seeds. All performance metrics were then calculated and reported as the mean ± 95% confidence interval (CI).

## 5. Conclusions

This study developed an early-warning framework for cucumber angular leaf spot in greenhouses by integrating low-cost particulate matter sensing, environmental monitoring, pathogen nucleic acid quantification, and disease index prediction. Evaluated across multiple spatially independent greenhouses, the ET-based surveillance model accurately estimated airborne pathogen concentrations, while the XGBoost-based prediction model effectively predicted the daily rate of disease index change. SHAP analysis identified airborne pathogen concentration, VPD, and temperature dynamics as key drivers of disease progression. The proposed pathogen-driven workflow provides earlier and more reliable risk assessment than approaches based only on symptoms or environmental conditions. By combining continuous sensor monitoring with risk-triggered qPCR validation, the system offers a scalable and practical strategy for real-time surveillance and precise management of airborne bacterial diseases in protected agriculture. Despite the high accuracy achieved, this study has certain limitations. The framework was validated for a single crop and pathogen in a limited number of greenhouses. Additionally, qPCR detects total pathogen DNA rather than exclusively viable cells, which may slightly overestimate the active infection risk. Future research should validate this system across diverse crops and open fields. Incorporating RNA detection or viability qPCR methods to directly identify living pathogens will overcome the limitations of total DNA quantification and significantly enhance disease forecasting precision.

## Figures and Tables

**Table 1:** Performance metrics of different pathogen aerosol surveillance models (Mean ± 95% CI).

Model Name	MAE (Mean ± 95% CI)	MAPE (Mean ± 95% CI)	R^2^ (Mean ± 95% CI)	RMSE (Mean ± 95% CI)
ET	0.332 ± 0.020	7.64% ± 0.29%	0.884 ± 0.007	0.454 ± 0.072
RF	0.403 ± 0.062	8.99% ± 0.24%	0.828 ± 0.010	0.567 ± 0.130
XGBoost	0.416 ± 0.068	9.21% ± 0.21%	0.797 ± 0.022	0.618 ± 0.190
DT	0.504 ± 0.062	11.08% ± 0.61%	0.620 ± 0.087	0.843 ± 0.299
LR	0.801 ± 0.104	18.56% ± 1.22%	0.345 ± 0.114	1.071 ± 0.259

Data are presented as mean ± 95% confidence interval (CI), derived from five independent modeling iterations. MAE, mean absolute error; MAPE, mean absolute percentage error; R^2^, coefficient of determination; RMSE, root mean square error; ET, Extra Trees; RF, Random Forest; XGBoost, Extreme Gradient Boosting; DT, Decision Tree; LR, Linear Regression.

**Table 2:** Performance of different models for predicting the disease index change rate (with 95% CI).

Model Name	MAE (Mean ± 95% CI)	MAPE (Mean ± 95% CI)	R^2^ (Mean ± 95% CI)	RMSE (Mean ± 95% CI)
XGBoost	0.095 ± 0.006	15.35% ± 1.98%	0.874 ± 0.028	0.191 ± 0.021
ET	0.097 ± 0.004	19.97% ± 1.10%	0.897 ± 0.020	0.174 ± 0.015
RF	0.113 ± 0.002	20.53% ± 0.39%	0.850 ± 0.016	0.213 ± 0.014
DT	0.098 ± 0.024	14.38% ± 7.19%	0.758 ± 0.113	0.252 ± 0.065
LR	0.344 ± 0.014	73.18% ± 3.13%	0.364 ± 0.040	0.453 ± 0.014

Data are presented as mean ± 95% confidence interval (CI), derived from five independent modeling iterations. MAE, mean absolute error; MAPE, mean absolute percentage error; R^2^, coefficient of determination; RMSE, root mean square error; ET, Extra Trees; RF, Random Forest; XGBoost, Extreme Gradient Boosting; DT, Decision Tree; LR, Linear Regression.

**Table 3:** Detailed information on the experimental greenhouses and plot allocations.

Greenhouse	Location (Coordinates)	Greenhouse Type	Trial Season	Dimensions (L × W × H, m)	Number of Experimental Plots
I	Haidian District, Beijing (116.33° E, 39.96° N)	Plastic arch tunnel	Spring, Autumn	36.27 × 13.60 × 3.74	6 (Spring), 6 (Autumn)
II	Haidian District, Beijing (116.33° E, 39.97° N)	Solar greenhouse	Spring	48.30 × 12.40 × 3.60	8
III	Pinggu District, Beijing (117.08° E, 40.19° N)	Solar greenhouse	Autumn	48.24 × 10.75 × 3.80	8

L, length; W, width; H, height.

## Data Availability

The data that support the findings of this study are available from the corresponding authors upon reasonable request. The data are not publicly available due to ongoing research utilizing this dataset for subsequent modeling studies.

## Abbreviations

The following abbreviations are used in this manuscript:

*Pal*	*Pseudomonas amygdali* pv. *lachrymans*
DI	Disease index
RMSE	Root mean square error
MAE	Mean absolute error
MAPE	Mean absolute percentage error
R^2^	Coefficient of determination
DT	Decision tree
XGBoost	Extreme gradient boosting
LR	Linear regression
VPD	Vapor pressure deficit
ET	Extra trees
RF	Random forest
SHAP	SHapley Additive exPlanations
VIF	Variance inflation factor
CI	Confidence interval
